# Supplementary material for: Resolution of RHCE Haplotype Ambiguities in Transfusion Settings
Source: Int J Mol Sci. 2024 May 28;25(11):5868. doi: 10.3390/ijms25115868 (PMC11172784; doi:10.3390/ijms25115868)
Supplement: Supplementary file 1 [file ijms-25-05868-s001.zip › Figure S7.pdf]

Supplementary Figure 7. mRNA and protein sequence of the new allele described in this study (*RHCE*cE_340T*; Genbank accession Number PP583668)

CTGGTGCTGGTGGAACCCCTGCACAGAGACGGACACAGGATGAGCTCTAAGTAC

CCGCGGTCTGTCCGGCGCTGCCTGCCCCTCTGCGCCCTAACACTGGAAGCAGCTC

TCATTCTCCTCTTCTATTTTTTTACCCACTATGACGCTTCCTTAGAGGATCAAAAG

GGGCTCGTGGCATCCTATCAAGTCGGCCAAGATCTGACCGTGATGGCGGCCCTTG

GCTTGGGCTTCCTCACCTCAAATTTCCGGAGACACAGCTGGAGCAGTGTGGCCTT

CAACCTCTTCATGCTGGCGCTTGGTGTGCAGTGGGCAATCCTGCTGGACGGCTTC

CTGAGCCAGTTCCCTCCTGGGAAGGTGGTCATCACACTGTTCAGTATTCGGCTGG

CCACCATGATTGCTATGTCGGTGCTGATCTCAGCGGGTGCTGTCTTGGGGAAGGT

CAACTTGGCGCAGTTGGTGGTGATGGTGCTGGTGGAGGTGACAGCTTTAGGCACC

CTGAGGATGGTCATCAGTAATATCTTCAACACAGACTACCACATGAACCTGAGGC

ACTTCTACGTGTTCGCAGCCTATTTTGGGCTGACTGTGGCCTGGTGCCTGCCAAAG

CCTCTACCCAAGGGAACGGAGGATAATGATCAGAGAGCAACGATACCCAGTTTG

TCTGCCATGCTGGGCGCCCTCTTCTTGTGGATGTTCTGGCCAAGTGTCAACTCTCC

TCTGCTGAGAAGTCCAATCCAAAGGAAGAATGCCATGTTCAACACCTACTATGCT

CTAGCAGTCAGTGTGGTGACAGCCATCTCAGGGTCATCCTTGGCTCACCCCCAAA

GGAAGATCAGCATGACTTATGTGCACAGTGCGGTGTTGGCAGGAGGCGTGGCTGT

GGGTACCTCGTGTCACCTGATCCCTTCTCCGTGGCTTGCCATGGTGCTGGGTCTTG

TGGCTGGGCTGATCTCCATCGGGGGAGCCAAGTGCCTGCCGGTGTGTTGTAACCG

AGTGCTGGGGATTCACCACATCTCCGTCATGCACTCCATCTTCAGCTTGCTGGGTC

TGCTTGGAGAGATCACCTACATTGTGCTGCTGGTGCTTCATACTGTCTGGAACGG

CAATGGCATGATTGGCTTCCAGGTCCTCCTCAGCATTGGGGAACTCAGCTTGGCC

ATCGTGATAGCTCTCACGTCTGGTCTCCTGACAGGTTTGCTCCTAAATCTCAAAAT

ATGGAAAGCACCTCATGTGGCTAAATATTTTGATGACCAAGTTTTCTGGAAGTTT

CCTCATTTGGCTGTTGGATTTTAAGCAAAAGCATCCAAGAAAAACAAGGCCTGTT

CAAAAACAAGACAACTTCCTCTCACTGTTGCCTGCATTTGTACGTGAGAAACGCT

CATGACAGCAAAGTCTCCTTATGTATAATGAAACAAGGTCAGAGACAGATTTGAT

ATTAAAAAATTAAAGACTAAAAACTTAGTTTAAGAGTCAATTTAATAAGTTTAAA

ATAAATGTTTAGTTTCATTAGGATGATGCTATCAATATTTTCTTGGTTACAGACAC

ATTATTAAAGTTTTGGGTTAATTTTA

MSSKYPRSVRRCLPLCALTLEAALILLFYFFTHYDASLEDQKGLVASYQVGQDLTVM
AALGLGFLTSNFRRHSWSSVAFNLFMLALGVQWAILLDGFLSQFPPGKVVITLFSIRL
ATMIAMSVLISAGAVLGKVNLAQLVVMVLVEVTALGTLRMVISNIFNTDYHMNLRH
FYVFAAYFGLTVAWCLPKPLPKGTEDNDQRATIPSLSAMLGALFLWMFWPSVNSPLL
RSPIQRKNAMFNTYYALAVSVVTAISGSSLAHPQRKISMTYVHSAVLAGGVAVGTSC
HLIPSPWLAMVLGLVAGLISIGGAKCLPVCCNRVLGIHHISVMHSIFSLLGLLGEITYIV
LLVLHTVWNGNGMIGFQVLLSIGELSLAIVIALTSGLLTGLLLNLKIWKAPHVAKYFD
DQVFWKFPHLAVGF
