# Supplementary material for: Resolution of RHCE Haplotype Ambiguities in Transfusion Settings
Source: Int J Mol Sci. 2024 May 28;25(11):5868. doi: 10.3390/ijms25115868 (PMC11172784; doi:10.3390/ijms25115868)
Supplement: Supplementary file 1 [file ijms-25-05868-s001.zip › Supplementary file captions_2979149.pdf]

Table S1. Primer characteristics used for *RHCE*-specific reverse transcription, amplification and bi-allelelic sequencing (specific positions are underlined in the primer sequences).

Table S2. Sequencing primer (forward, exon 2) characteristics used for *RHCE**c and *RHCE*C* specific analysis (specific positions are underlined in the primer sequences).

Table S3. Description of samples used to validate the *RHCE* mRNA analysis protocol . RhD and RhC phenotypes, i.e. presence (+) or absence (-) of Rh antigens (RH1 (D), RH2 (C), RH3 (E), RH4 (c), RH5 (e)), and *RHCE* haplotypes are given.

Figure S1. Reticulocyte isolation purity assessment using flow cytometry. The cell population was detected according to its morphology using an initial gate set in an FSC-A/SSC-A plot; single cells were further gated in an FSC-A/FSC-H plot; unstained (A) and isotype staining (B) were gated in a subsequent FITC-A/SSC-A plot and compared to cells expressing CD71 (C).

Figure S2. (a) *ACTB* (actinβ) mRNA and (b) *18S* ribosomal RNA real-time PCR analysis (absolute Ct) according to the time between blood collection and processing at 24 hours, 36 hours, 48 hours and seven days after collection for the antigen-specific isolation of reticulocytes using three separation columns (Ordinary one-way ANOVA, Multiple comparisons. One asterisk (*) indicates p value smaller than 0.05 ($p<0.05$); three asterisks (***) indicate p value smaller than 0.001 ($p<0.001$); four asterisks (****) indicate p value smaller than 0.0001 ($p<0.0001$)).

Figure S3. Example of specific *RHCE* sequencing as regards to *RHD* of an RhDCe sample. *RHD* and *RHCE* text sequences are aligned with the sample sequenced with the RHCE-seq-R1 primer (Table S1, specific for both *RHCE* haplotypes). Vertical lines highlight the positions c.361 (*RHD*>T; *RHCE*>A), c.380 (*RHD*>T; *RHCE*>C), c.383 (*RHD*>A; *RHCE*>G) and c.455 (*RHD*>A; *RHCE*>C)).

Figure S4. Example of RHCE haplotype-specific sequencing of a *RHCE*01/*04* sample. The chromatograms highlight the c.676 G>C polymorphism (vertical line). RT-PCR products were sequenced (A) with the *RHCE*-seq-R1 primer (Table S1, specific for both *RHCE* haplotypes) and show the heterozygous signal for c.676 and (B) with the RHc_ex2_178a203F primer (Table S2, specific for *RHc* haplotype) and show the hemizygous signal for c.676 G (corresponding to the *RHCE*01* haplotype).

Figure S5. Confirmation of the *RHCE*02/RHCE*03.13* genotype. The chromatograms highlight the c. 676G>C and the c.728A>G polymorphisms (vertical lines). RT-PCR products were sequenced with (A) the RHc_ex2_178a203F primer (Table S2, specific for *RHc* haplotype) and show the hemizygous signal for c.676C and c.728G (corresponding to the *RHCE*02* haplotype) and (B) the RHgrandC_ex2_178a203F (Table S2, specific for *RHC* haplotype) and show the hemizygous signal for c.676G and c.728A (corresponding to the *RHCE*03.13* haplotype).

Figure S6. Identification of the novel allele *RHCE*03* c.340C>T. The chromatograms highlight the c.307C>T and the c.340C>T polymorphisms (vertical lines). RT-PCR products were sequenced (A) with the RHgrandC_ex2_178a203F (Table S2, specific for *RHC* haplotype) and show the hemizygous signal for c.307T and c.340C (corresponding to the *RHCE*02* allele) and (B) with the RHc_ex2_178a203F primer (Table S2, specific for *RHc* haplotype) and show the hemizygous signal for c.307C and c. c.340T (corresponding to the *RHCE*03 c.340T* allele).

Figure S7. mRNA and protein sequence of the novel allele described in this study (*RHCE*cE_340T*; Genbank accession Number PP583668)
