# Supplementary material for: Resolution of RHCE Haplotype Ambiguities in Transfusion Settings
Source: Int J Mol Sci. 2024 May 28;25(11):5868. doi: 10.3390/ijms25115868 (PMC11172784; doi:10.3390/ijms25115868)
Supplement: Supplementary file 1 [file ijms-25-05868-s001.zip › Table S1 S2 S3_2979149_R1.pdf]

Table S1. Primers' characteristics used for *RHCE*-specific reverse transcription, amplification and bi-allelelic sequencing (specific positions are underlined in the primer sequences).

Name	specificity	Location	Application	Sens	Sequence
RHCE-PCRex7R	*RHCE*	Exon 7	RT & PCR	Reverse	AAGATGGAGTGCATGACGGA
RHDCE-PCRex1F	*RHD*/*RHCE*	Exon 1	PCR	Forward	ATGAGCTCTAAGTACCCGCG
RHCE-seq-R1	*RHCE*	Exon 5	Sequencing	Reverse	CATGCTGATCTTCCTTTGGGG

Table S2. Sequencing primers (forward, exon 2) characteristics used for *RHCE**c and *RHCE*C* specific analysis (specific positions are underlined in the primer sequences).

Name	Sequence	Specificity	Specific positions
RHc_ex2_178a203F	CTTGGCTTGGGCTTCCTCACCTCAAA	*RHCE**c	c.178G, c.201A, c.203A
RHgrandC_ex2_178a203F	ATTGGCTTGGGCTTCCTCACCTCGAG	*RHCE*C*	c.178A, c.201G, c.203G

Table S3. Samples description used for *RHCE* mRNA analysis protocol validation. RhD and RhC phenotypes, i.e. presence (+) or absence (-) of Rh antigens (RH1 (D), RH2 (C), RH3 (E), RH4 (c), RH5 (e)), and *RHCE* haplotypes are given.

Number of samples	D RH1	C RH2	E RH3	c RH4	e RH5	*RHCE* haplotypes
13	+	-	-	+	+	*RHCE*ce (RHCE*01)/RHCE*ce (RHCE*01)*
11	+	+	-	+	+	*RHCE*ce (RHCE*01)/RHCE*Ce (RHCE*02)*
1	+	-	+	+	+	*RHCE*ce (RHCE*01)/RHCE*cE (RHCE*03)*
1	+	+	-	-	+	*RHCE*Ce (RHCE*02)/ RHCE*Ce (RHCE*02)*
4	+	+	+	+	+	*RHCE*Ce (RHCE*02)/RHCE*cE (RHCE*03)*
