# Supplementary figures and images for: Resolution of RHCE Haplotype Ambiguities in Transfusion Settings
Source: Int J Mol Sci. 2024 May 28;25(11):5868. doi: 10.3390/ijms25115868 (PMC11172784; doi:10.3390/ijms25115868)

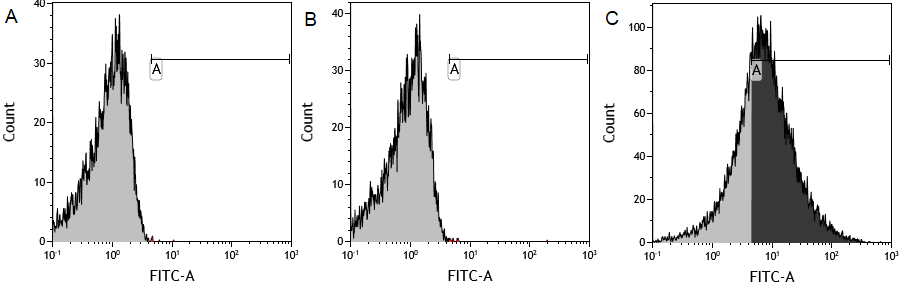

Supplement: Supplementary file 1 [file ijms-25-05868-s001.zip › FigureS1.tif]

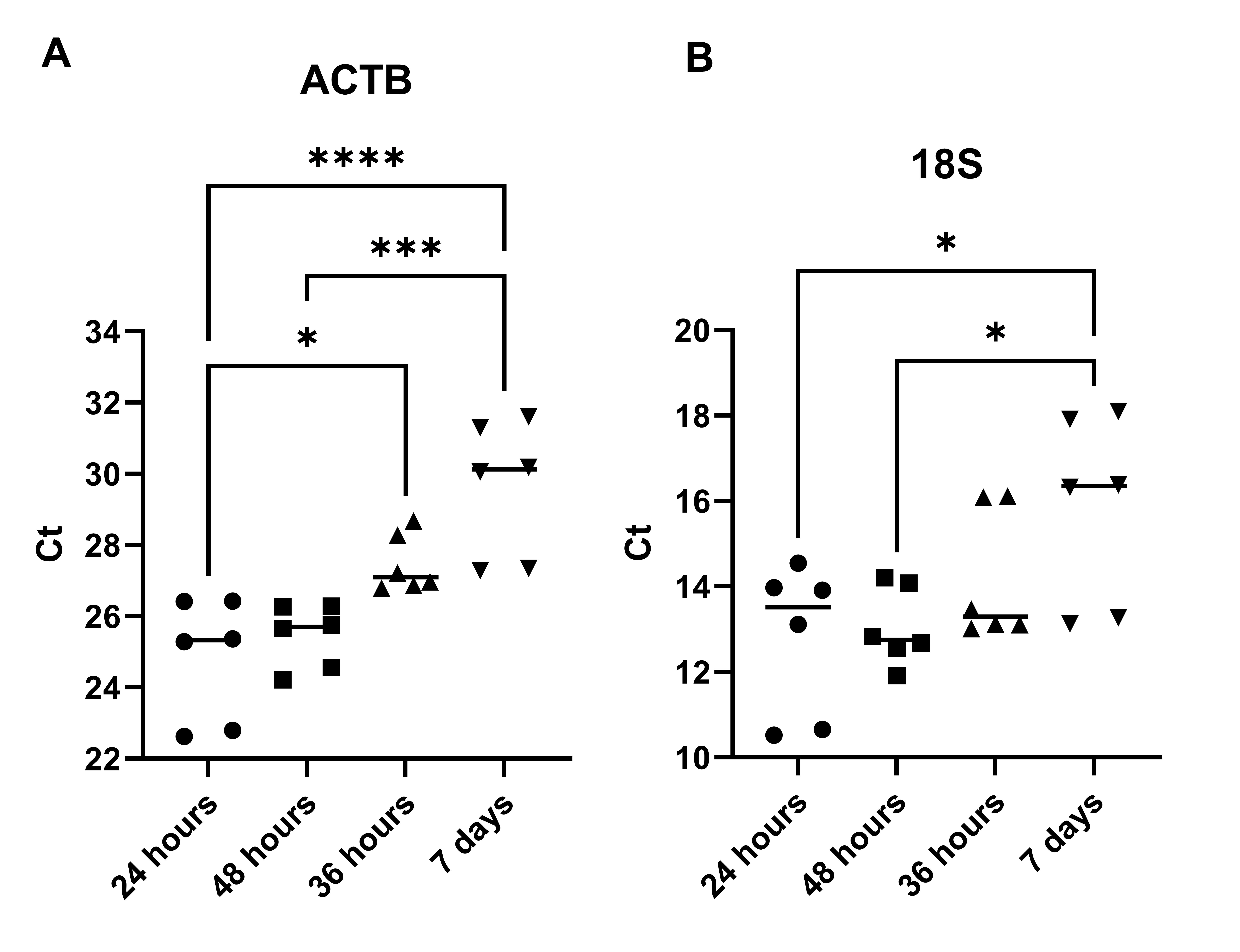

Supplement: Supplementary file 1 [file ijms-25-05868-s001.zip › FigureS2.tif]

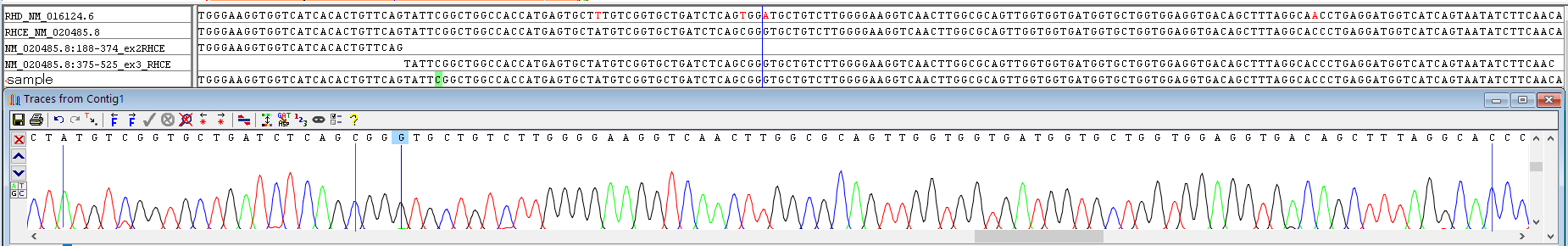

Supplement: Supplementary file 1 [file ijms-25-05868-s001.zip › FigureS3.tif]

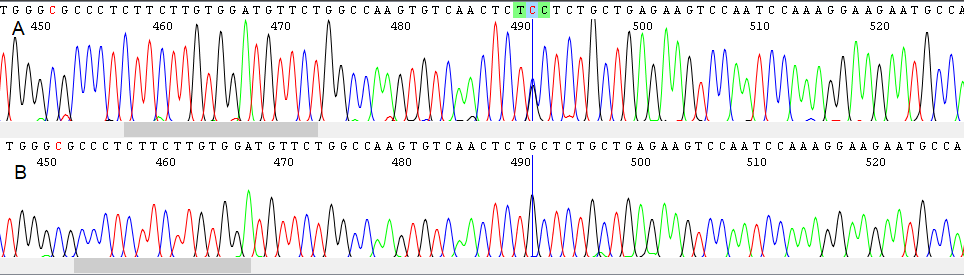

Supplement: Supplementary file 1 [file ijms-25-05868-s001.zip › FigureS4.tif]

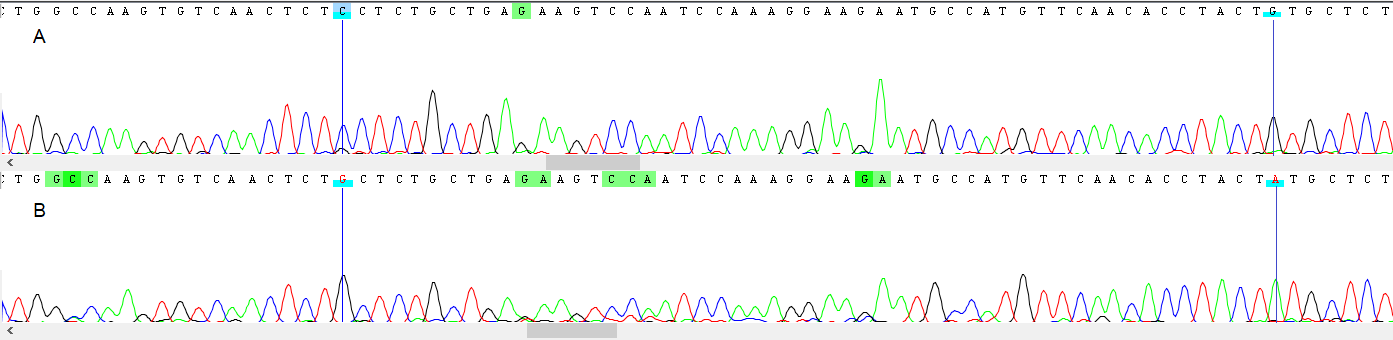

Supplement: Supplementary file 1 [file ijms-25-05868-s001.zip › FigureS5.tif]

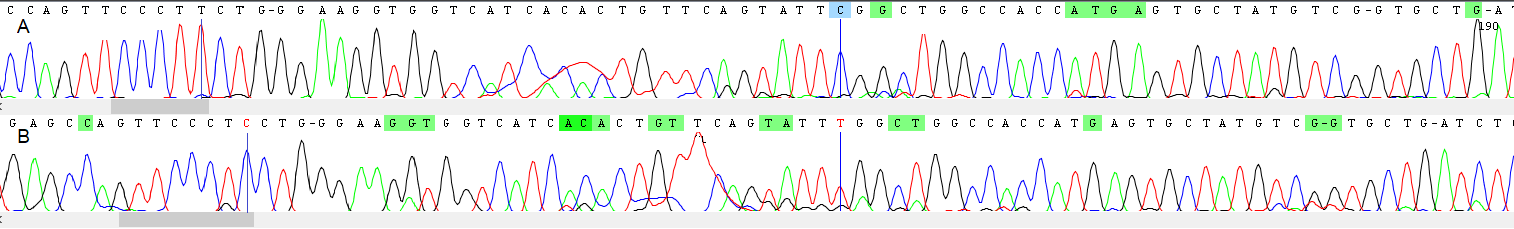

Supplement: Supplementary file 1 [file ijms-25-05868-s001.zip › FigureS6.tif]
